# Evaluation of the Effects of Isoflurane Anaesthesia on Gastrointestinal Transit Time, Feed Intake and Defecation in Rabbits

**DOI:** 10.1002/vms3.71272

**Published:** 2026-10-30

**Authors:** Amir Zarein, Amir Rostami, Majid Masoudifard, Saeed Farzad‐Mohajeri, Alireza Bahonar, Haniyeh Naghdi Liyeh, Zahra Sohani

**Affiliations:** ^1^ Department of Veterinary Internal Medicine Faculty of Veterinary Medicine University of Tehran Tehran Iran; ^2^ Department of Surgery and Radiology Faculty of Veterinary Medicine University of Tehran Tehran Iran; ^3^ Division of Epidemiology & Zoonoses Department of Food Hygiene and Quality Control Faculty of Veterinary Medicine University of Tehran Tehran Iran

**Keywords:** contrast radiography, gastrointestinal motility, gastrointestinal transit, hindgut fermenters, isoflurane, postoperative complications, rabbit anaesthesia

## Abstract

**Background:**

Domestic rabbits are popular companion animals, resulting in a growing demand for veterinary procedures requiring anaesthesia. However, due to their unique characteristics, anaesthetizing rabbits is challenging and carries a high risk of peri‐anaesthetic mortality and gastrointestinal (GI) complications. Isoflurane is a commonly used inhalation anaesthetic, but no studies have assessed its potential effects on GI transit time in rabbits.

**Objectives:**

The present study aims to assess whether varying durations of isoflurane anaesthesia affect GI transit time, feed intake and defecation in healthy rabbits.

**Methods:**

Five healthy adult male New Zealand White rabbits participated in a repeated measures study comprising four conditions: (1) awake (control), (2) 15 min, (3) 30 min and (4) 60 min of anaesthesia with isoflurane, each separated by a 6‐day washout period. Anaesthesia was induced with 5% isoflurane in 100% oxygen using an induction chamber and maintained with 1.5%–3% isoflurane in oxygen delivered via an endotracheal tube to achieve deep surgical anaesthesia. GI transit time was evaluated via barium sulphate contrast radiography at 1, 15, 30, 60, 120, 240, 360 and 420 min post‐administration. The study assessed three transit markers: (1) gastric transit time, (2) small intestinal transit time and (3) time to hard faeces formation. Feed intake and faecal output were also recorded daily for 6 days after each anaesthetic condition.

**Results:**

Gastric transit time was consistent across all experimental conditions, and the contrast agent was seen at T1 minute across all conditions. Small intestinal transit time differed significantly among groups (Friedman test: *χ* ^2^ (3) = 10.41, *p* = 0.015, Kendall's *W* = 0.69); however, pairwise comparisons between anaesthesia conditions and the awake control were not statistically significant. The 60‐min group had the highest median transit time (120 min), compared with 60 min in the other three conditions. No significant differences were observed in time to hard faeces formation among conditions (*p* = 0.152). Feed intake and faecal output did not differ across anaesthetic durations (*p* > 0.05 for all comparisons).

**Conclusions:**

Under the experimental conditions studied, isoflurane anaesthesia was not associated with significant changes in gastric transit, the time to hard faeces formation, feed intake or faecal output in healthy male rabbits. Although we observed an overall difference in small intestinal transit time, no anaesthetic group showed a statistically significant variation from the awake control phase. Furthermore, the discrete nature of radiographic assessments and the small sample size limit definitive conclusions regarding a clinically relevant isoflurane‐induced transit delay. Further studies with larger sample sizes and more precise transit‐time measurement techniques are recommended.

## Graphical abstract

Isoflurane anaesthesia was evaluated for its effects on gastrointestinal transit time, feed intake and faecal output in five healthy male rabbits. No clinically evident changes were observed in gastric transit, formation of hard faeces, feed intake or faecal output. However, small‐intestinal transit time showed a significant difference that warrants cautious interpretation.

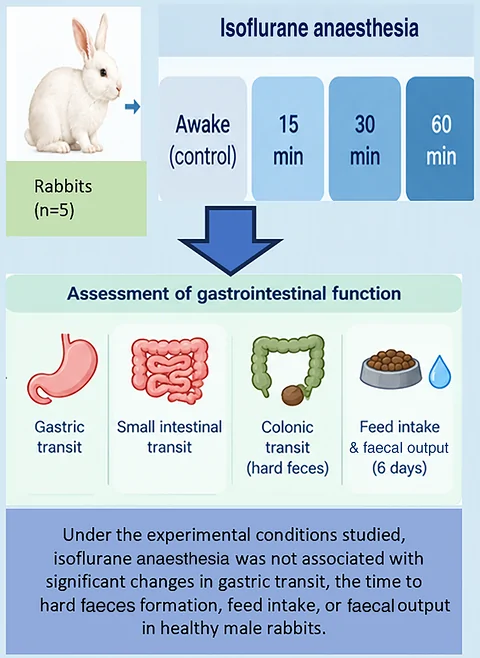

## 1 Introduction

Domestic rabbits are increasingly popular as companion animals, resulting in a growing demand for veterinary procedures that require anaesthesia, whether for surgical or diagnostic interventions (Wenger 2012). However, the unique anatomical, physiological and behavioural characteristics of rabbits make anaesthesia particularly challenging, leading to a significantly higher risk of peri‐anaesthetic mortality and gastrointestinal (GI) complications when compared to cats and dogs. In fact, a retrospective study indicated that 38% of anaesthetic events were attributed to peri‐anaesthetic GI complications (Lee et al. 2018).

As hindgut fermenters, rabbits depend on regular GI motility to maintain proper digestive function (Oglesbee and Lord 2020). Disruption of this delicate system can lead to a condition known as GI stasis syndrome, characterized by reduced or absent GI motility. GI stasis can result in blockages and the proliferation of harmful bacteria, which may manifest as symptoms like hyporexia, reduction of faecal production and abdominal discomfort (Bellier and Gidenne 1996).

Anaesthesia is well‐known for its influence on GI physiology across different species (Lee et al. 2018). Isoflurane—a commonly used inhalation anaesthetic—has gained popularity due to its rapid induction and recovery times, as well as its minimal biotransformation compared to other anaesthetics (Flecknell 2009). These qualities make isoflurane suitable for a variety of experimental procedures, ranging from brief interventions to more extensive surgical operations. However, no studies have assessed the potential effects of isoflurane on GI transit time in rabbits (Feldman et al. 2021).

Given these concerns, the present study aims to assess whether varying durations of isoflurane anaesthesia affect GI transit time, feed intake and defecation patterns in healthy rabbits. Considering the rabbit's highly sensitive GI system and the relatively common occurrence of post‐anaesthetic digestive complications, the results of this research could help refine anaesthetic protocols and improve perioperative care for this species.

## 2 Materials and Methods

Six healthy adult male New Zealand White rabbits were initially enrolled in the study. One rabbit was excluded during the experimental period after an unforeseen incident involving accidental ingestion of the tube used in the procedure. Therefore, five rabbits completed the full experimental protocol and were included in the final statistical analysis. The five rabbits in the analysis were 8–12 months old and weighed 3–3.5 kg. Rabbits were housed individually in cages with linear slotted floors under controlled environmental conditions (16–22°C, 30%–70% relative humidity, 12‐h light/dark cycle) and underwent a 6‐day washout period before the study. Animals received 200 g/day of commercial pellets (Gidenne et al. 2010) containing 85% forage and 15% alfalfa hay, with ad libitum access to fresh water. Cages were cleaned daily with water. During the study and washout period, pellets and faeces were collected and weighed daily at 09:00.

### 2.1 Study Design

The study evaluated whether varying durations of isoflurane anaesthesia induce acute or persistent effects on GI transit time, feeding behaviour and defecation patterns in rabbits. A repeated measures design was employed, with each rabbit serving as its own control. Four experimental conditions were applied to each rabbit, with a 6‐day interval between conditions: (1) Awake (control), (2) 15 min of anaesthesia, (3) 30 min of anaesthesia and (4) 60 min of anaesthesia.

During each study period, GI transit time was assessed using a standardized method (see GI transit time section for details). After evaluation in the awake phase (control), the order of experimental conditions was randomized for each rabbit using Excel v2021 (Microsoft Corporation, Redmond, WA, USA) to minimize potential order effects. A 6‐day washout period was implemented between conditions to eliminate carry‐over effects and allow for physiological normalization. Throughout each 6‐day interval, daily measurements of food intake and faecal output (in grams) were recorded for each rabbit.

### 2.2 Anaesthesia and Monitoring

Food was not withheld from rabbits before anaesthesia. Anaesthesia was induced at 10:00 with 5% isoflurane (Terrell, Piramal Critical Care, Bethlehem, PA, USA) in 100% oxygen using an induction chamber (Figure 1). Rabbits were removed from the chamber when the righting reflex was absent, indicating adequate sedation. An uncuffed, 2.5 mm endotracheal tube (ETT) (Bever, Hangzhou BEVER Medical Devices, Hangzhou, China) was then inserted using a blind orotracheal intubation technique. Proper ETT placement was confirmed by capnography, after which the rabbit was connected to a compact breathing system (COSY) designed for low‐flow ventilation (Dräger Fabius, Dräger, Lübeck, Germany). Anaesthesia was maintained with 1.5%–3% isoflurane in oxygen delivered via the ETT to achieve deep surgical anaesthesia, as determined by muscle relaxation and absence of palpebral and corneal reflexes. Experimental durations of 15, 30 and 60 min were defined as the elapsed time from the establishment of deep surgical anaesthesia, following ETT placement. Rabbits were positioned laterally throughout the procedure, and both eyes were kept moist with ophthalmic ointment (NAJO‐TEARS; Iran Najo, Karaj, Iran). Physiological parameters, including heart rate, pulse rate, oxygen saturation (SpO2), respiratory rate and end‐tidal carbon dioxide (ETCO2), were monitored throughout anaesthesia using a vital signs monitor (Cardioset ARAD P10, SAIran Med, Isfahan, Iran) (Figure 2). The procedure concluded when the anaesthetic gas was discontinued. Rabbits then received 100% oxygen, and the ETT was removed once coughing or chewing reflexes returned. Upon regaining full consciousness, rabbits were prepared for the GI transit time study.

**FIGURE 1:**
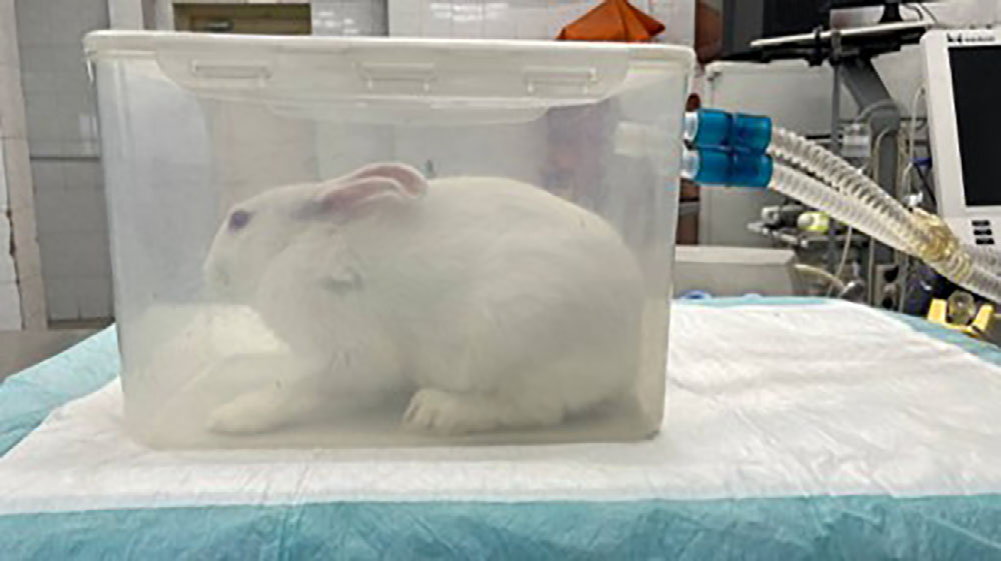
Anaesthesia induced with isoflurane 5% in 100% oxygen using an induction chamber.

**FIGURE 2:**
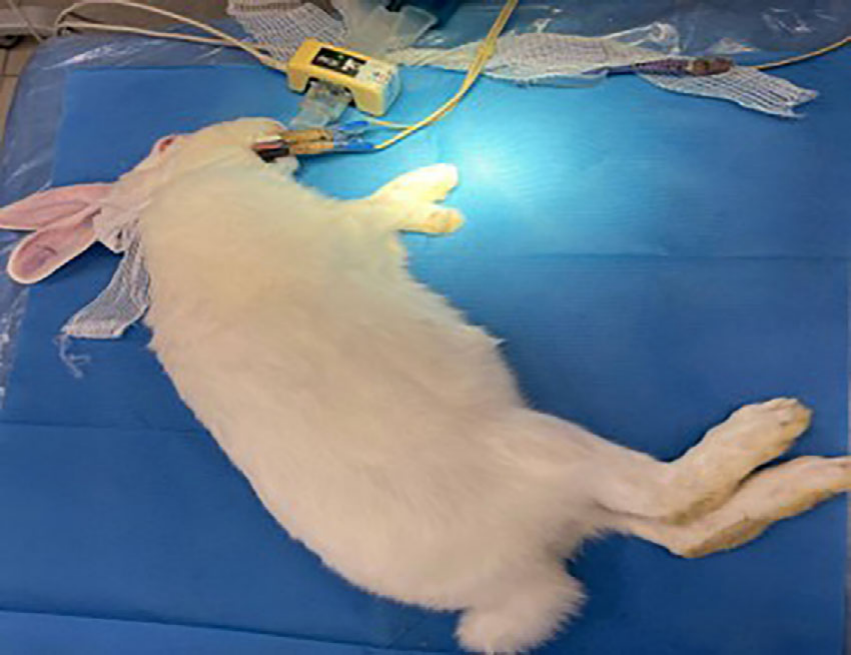
Anaesthesia was maintained with 1.5%–3% isoflurane via endotracheal tube. Heart rate was monitored using a pulse oximeter.

### 2.3 Assessment of GI Transit Time

GI transit time was initially evaluated in awake rabbits to establish a control model. The effects of isoflurane on GI transit time were then assessed under anesthetized conditions. At each study point, the distance from the last rib to the nostril was measured to determine the appropriate nasogastric tube length (Brown 2010). Before tube insertion, the nostril was anesthetized with 2% topical lidocaine gel (Xylogel, Sina Darou, Tehran, Iran), and the nasogastric tube (SUPA Medical Devices, Karaj, Iran) was inserted through the medial aspect of the nostril (Figure 3.1). Systemic analgesics or tranquilizers were not administered to avoid confounding effects. Tube placement was confirmed using a lateral radiograph (Figure 3.2). A 30% barium sulphate suspension (12 mL/kg) (Barex, Darou Pakhsh Pharmaceutical, Tehran, Iran) (Jekl 2013) was then administered via the nasogastric tube. Radiographs were taken at 1, 15, 30, 60, 120, 240, 360 and 420 min after barium administration to assess contrast agent transit (Jekl 2013). The study was concluded when barium was observed in firm faecal matter within the colon. All radiographs were obtained at 55 kVp, 4.00 mAs (Toshiba Rotanode, Toshiba Medical Systems, Tokyo, Japan) in ventro‐dorsal and right latero‐lateral positioning. Three markers were defined for transit evaluation (Botman et al. 2020) (Figure 4). (1) Gastric transit time: the time at which barium first appeared in the small intestine. (2) Small intestinal transit time: the time at which barium first appeared in the caecum. (3) Time to hard faeces formation: the time at which barium was first detected in solid faeces within the colon.

**FIGURE 3:**
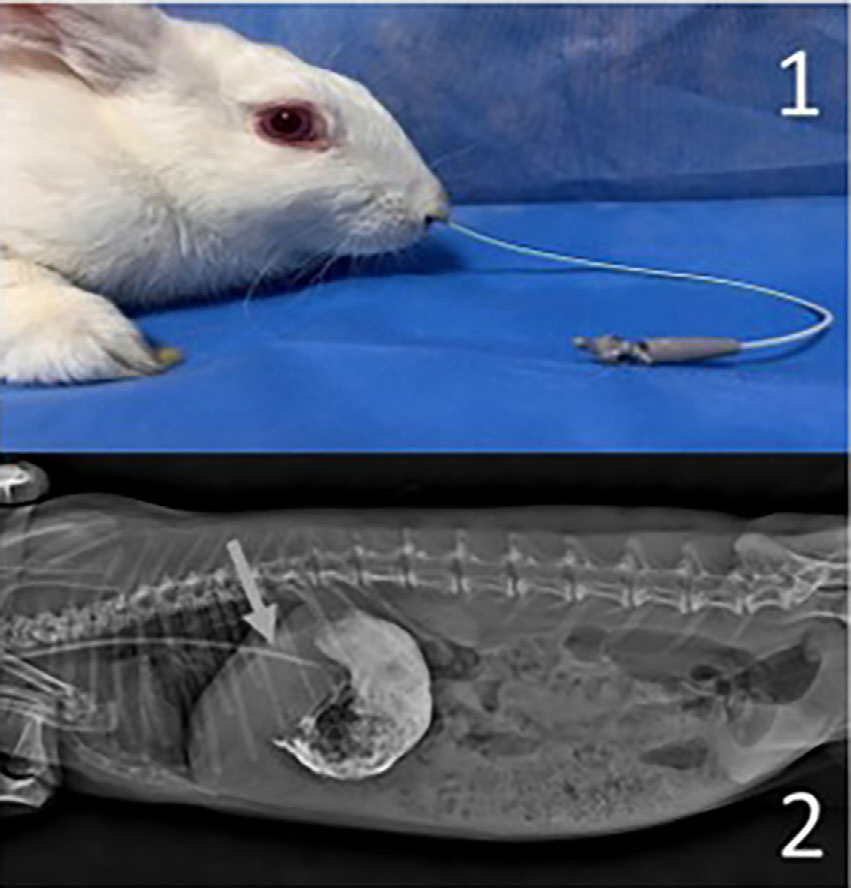
(1) Nasogastric tube insertion for administering barium sulphate (2) tube placement was confirmed using a lateral radiograph (arrow).

**FIGURE 4:**
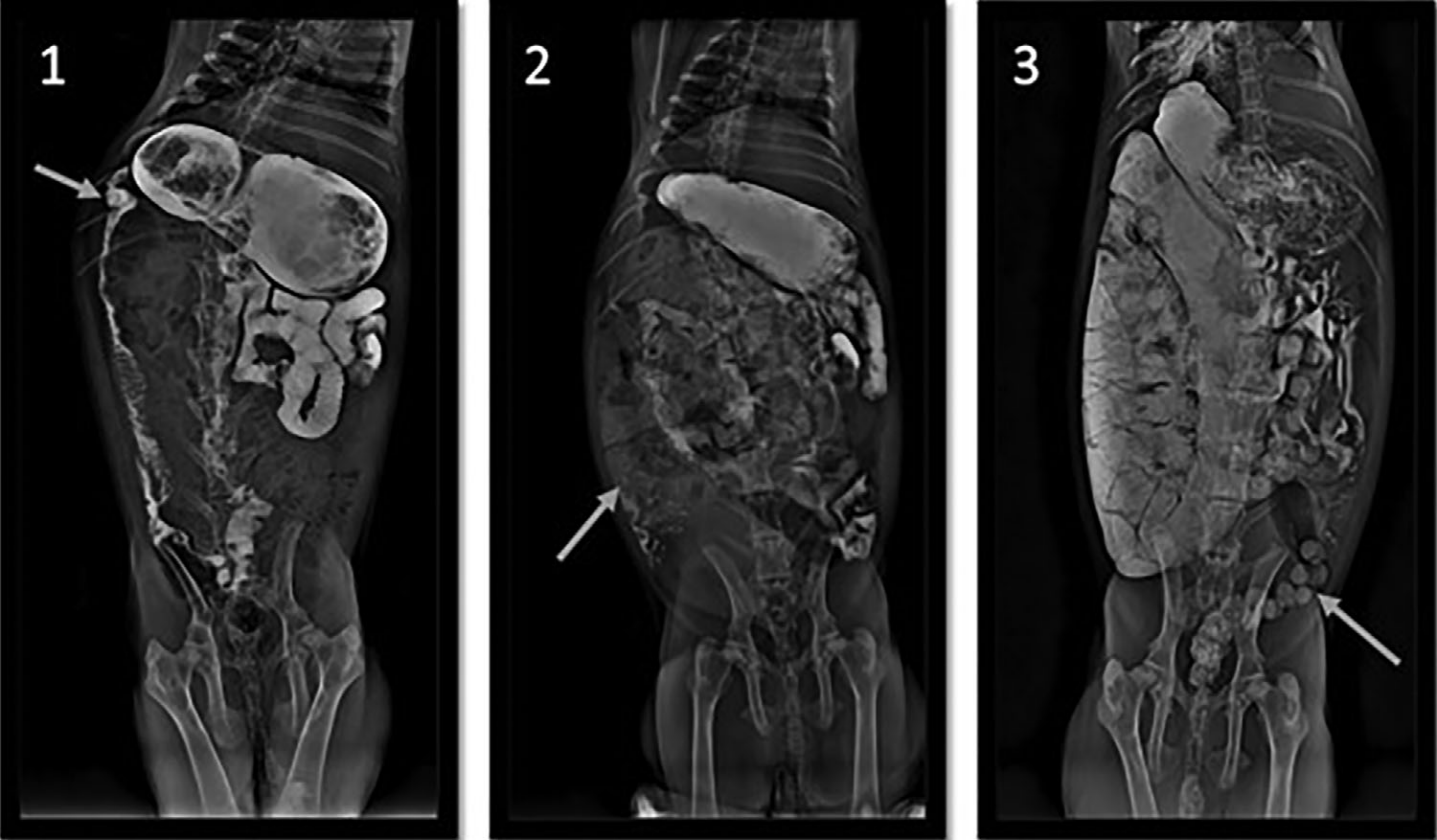
Assessment of gastrointestinal transit time. The time at which contrast agent was detected in the small intestine (1), caecum (2) and as hard faeces in the colon (3) were called, respectively, gastric transit time, small intestine transit time and time to formation of hard faeces.

### 2.4 Measurement of Faecal Output and Feed Intake

Feed and faeces measurements were recorded daily at 09:00. Feed intake was calculated by subtracting the weight of the remaining pellets from the initial 200 g provided over the previous 24 h. To minimize the impact of moisture on faecal weight, paper towels were placed in the defecation area beneath the cages to absorb urine present in the container. The total faecal output was collected, weighed and recorded.

### 2.5 Statistical Analysis

Data were organized using Excel v2021 (Microsoft Corporation, Redmond, Washington, USA). Normality was assessed with the Shapiro–Wilk test. Parametric data were analysed using two‐way repeated‐measures ANOVA, with effect sizes reported as partial eta squared (partial *η* ^2^). Nonparametric data were analysed using the Friedman test, with Kendall's *W* reported as the effect size for overall comparisons. In cases where the Friedman test revealed significant differences, pairwise comparisons were carried out using the Wilcoxon signed‐rank test, with effect sizes calculated as *r* = |*Z*|/√*N*. Ninety‐five per cent confidence intervals (CIs) for the mean were reported to indicate the precision of the estimates. Statistical significance was set at *α* = 0.05. All analyses were performed using SPSS Statistics version 27 (IBM Corporation, Armonk, NY, USA).

## 3 Results

### 3.1 GI Transit Time

Results of GI transit time are summarized in Table 1. Nonparametric methods were employed due to deviation from normality as indicated by the Shapiro–Wilk test. Gastric transit time remained consistent, and the contrast agent was seen at T1 minute across all conditions; therefore, no statistical comparison was applicable. In contrast, small intestinal transit time showed a significant overall difference among groups (Friedman test: *χ* ^2^ (3) = 10.41, *p* = 0.015, Kendall's *W* = 0.69). The median for small intestinal transit times was 60 min (range, 30–120 min) for the awake, 15‐min and 30‐min groups, and 120 min (range, 60–120 min) for the 60‐min group. Pairwise comparison using the Wilcoxon signed‐rank test revealed statistically significant differences between the 30‐ and 60‐min anaesthesia groups (*p* = 0.038, *r* = 0.92). No other pairwise comparison achieved statistical significance. Time to hard faeces formation did not differ significantly among conditions (Friedman test: *χ* ^2^ (3) = 5.286, *p* = 0.152, Kendall's *W* = 0.35).

**TABLE 1:** Mean (SD) (95% confidence interval [CI]) gastrointestinal transit times in five rabbits in the awake state and 15, 30 and 60 min after anaesthesia with isoflurane.

	Awake	15 min	30 min	60 min
Gastric transit time (min)	1(0) (1–1)	1(0) (1–1)	1(0) (1–1)	1(0) (1–1)
Small intestine transit time (min)	60(0) (60–60)	84(32.86) (43–124)	48(16.43) (27–68)	96(32.86) (55–136)
Time to hard faeces formation (min)	168(65.7) (86–249)	240(0) (240–240)	180(84.9) (74–285)	192(65.7) (110–273)

### 3.2 Feed Intake and Faecal Output

Data from feed consumption and faecal output are summarized in Table 2. Two‐way repeated‐measures ANOVA revealed no statistically significant main effects of anaesthesia group (*F*(1.65, 6.59) = 0.553, *p* = 0.567, partial *η* ^2^ = 0.121), time (*F*(1.93, 7.71) = 2.127, *p* = 0.184, partial *η* ^2^ = 0.347) or the interaction between group and time (*F*(2.13, 8.53) = 1.075, *p* = 0.387, partial *η* ^2^ = 0.212) in feed intake. Analysis of the daily faecal output also showed no significant main effects for anaesthesia group (*F*(2.05, 8.20) = 1.126, *p* = 0.330, partial *η* ^2^ = 0.242), time (*F*(2.55, 10.18) = 1.332, *p* = 0.313, partial *η* ^2^ = 0.250) or the interaction effect (*F*(3.57, 14.27) = 1.491, *p* = 0.258, partial *η* ^2^ = 0.272).

**TABLE 2:** Mean (SEM) (95% confidence interval [CI]) of the feed intake and faecal output in five rabbits during 6 days in awake state and after 15, 30 and 60 min of anaesthesia with isoflurane.

	Feed intake	Faecal output
Adaptation (g)	100.26(0.59) (98–101)	51.60(1.48) (47–55)
Awake (g)	100.36(0.53) (98–101)	51.83(1.15) (48–55)
15 min of anaesthesia (g)	99.93(0.51) (98–101)	52.46(1.77) (47–57)
30 min of anaesthesia (g)	100.56(0.29) (99–101)	50.03(1.49) (45–54)
60 min of anaesthesia (g)	99.00(1.65) (94–103)	49.20(2.31) (42–55)

## 4 Discussion

This study represents the first assessment of isoflurane's effects on GI transit in rabbits. The omnibus Friedman test demonstrated a significant overall difference in small intestinal transit time among conditions (*p* = 0.015) without any significant changes in gastric transit, time to hard faeces formation or post‐anaesthetic daily feed intake and faecal output. Although the 60‐min anaesthesia group had the highest median small intestinal transit time (120 min), compared with 60 min in the awake, 15‐min and 30‐min groups, the difference between the 60‐min and awake control groups was not statistically significant (*p* = 0.083). No other anaesthesia group differed significantly from the awake control. Therefore, the present findings do not provide sufficient evidence to conclude that isoflurane causes a clinically relevant delay in small intestinal transit time. The statistically significant difference observed between the 30 and 60‐min groups (*p* = 0.038) warrants cautious interpretation. The significant pairwise comparison does not independently confirm a duration‐dependent effect of isoflurane, especially considering that neither group exhibited a significant difference from the awake control. The 30‐min group demonstrated the shortest transit time (mean 48.00 (SEM 16.43)), which was 12 min faster than the awake (control) group; however, this difference was not statistically significant (*p* = 0.157). Given the small sample size (*n* = 5), the discrete timing of radiographic sampling and substantial inter‐individual variability, this numerical difference in small intestinal transit time should not be interpreted as evidence of an actual pharmacological effect of isoflurane. As radiographs were obtained at fixed intervals, a recorded transit time of T60 minutes signifies that barium reached the caecum between the T30 and T60 minute radiographs, rather than precisely at T60 minutes. Although this approach is standard in rabbit contrast radiography (Jekl 2013), it reduces measurement precision and produces discrete ordinal data. Therefore, the observed statistically significant difference in transit time between the 30‐ and 60‐min groups (*p* = 0.038) may have been influenced by the discrete data collection, rather than an actual change in GI physiology. More frequent or continuous assessment of GI transit time would be required to determine whether the observed difference represents a reproducible effect of isoflurane.

In the absence of rabbit‐specific data, extrapolations from studies in other species can provide valuable insights into interpreting our findings. Torjman et al. (2005) examined the effects of isoflurane on GI motility in rats, demonstrating that short exposures (less than 6 min) have a time‐dependent impact on post‐anaesthetic GI function, with significant recovery observed after 120 min post‐anaesthesia. They reported that even continuous isoflurane anaesthesia for 120 min does not completely suppress motility in rats (Torjman et al. 2005). We also observed that even 60 min of isoflurane anaesthesia in rabbits did not lead to prolonged post‐anaesthetic complications. In another study in horses undergoing arthroscopic surgery, isoflurane anaesthesia was associated with a faster return to normal intestinal motility compared with halothane (Durongphongtorn et al. 2006). Although motility in that study was assessed by auscultation rather than imaging or contrast‐based techniques, their results support the relative safety of isoflurane in preserving postoperative GI function. These cross‐species findings, along with our data, support the conclusion that isoflurane's influence on GI motility is transient, and its effects do not extend beyond the immediate postoperative period.

A notable observation in this study was the apparent regional pattern, which was limited to small intestinal transit time, with no significant changes in gastric emptying or time to hard faeces formation. Dynamic MRI studies have shown that, during isoflurane anaesthesia in rats, jejunal contractions are markedly suppressed, with a concurrent reduction in the propagation of intestinal contents (Ailiani et al. 2014). Although similar studies have not been conducted in rabbits, ultrasonographic investigations indicate that measurable peristaltic activity in sedated and non‐sedated rabbits is primarily confined to the duodenum and jejunum, rather than the stomach, caecum or colon (Oura et al. 2019). This observation highlights the diagnostic value of duodenal motility as a reliable indicator of GI function in rabbits (Fitzmaurice et al. 2025). Although the current methodology does not allow for the precise determination of which segment of the small intestine is most influenced by isoflurane, the present contrast radiography study demonstrated that transit alterations following isoflurane anaesthesia were most pronounced in the small intestine. This suggests that the small intestine is the most functionally dynamic region and may be more sensitive to isoflurane‐induced suppression. However, no significant pairwise differences in small intestinal transit time were observed between anaesthetic groups and awake controls, limiting the ability to draw definitive conclusions regarding segment‐specific effects. Consequently, although these findings align with regional differences reported in the literature, definitive conclusions about segment‐specific sensitivity to isoflurane in rabbits will require further studies employing complementary methodologies.

At the cellular level, previously reported effects of isoflurane may provide potential hypotheses for the observed findings. Isoflurane induces the generation of reactive oxygen species (ROS) within cells, leading to ROS accumulation that subsequently impacts mitochondrial function (Simon et al. 2000; Sun et al. 2014). This dysfunction promotes a shift away from the contractile phenotype and, indirectly, impairs smooth muscle contractility (Pearce 2024). Sun et al. (2014) reported time‐dependent ROS accumulation following isoflurane exposure in human embryonic kidney (HEK‐293) cells, with increased ROS levels observed after 60 and 90 min but not after 15 or 30 min (Sun et al. 2014). The authors also suggested that isoflurane‐induced ROS accumulation may not be restricted to a specific cell type. These findings cannot be directly extrapolated to GI smooth muscle in rabbits. Notably, the present study did not evaluate ROS production, mitochondrial function or smooth muscle contractility, and thus cannot establish a causal relationship between these mechanisms and GI transit. The 60‐min anaesthesia group had the longest small intestinal transit time (mean 96 [SEM 14.69]) and a higher median (120 min) compared to the other conditions. However, the post hoc pairwise comparison between the 60‐min group and the control group did not reach statistical significance. Consequently, any association between prolonged isoflurane exposure and altered GI motility, including a potential cellular mechanism involving oxidative stress or a potential biphasic dose‐response relationship, remains speculative and warrants direct investigation.

The key strength of this study is its non‐surgical design. Pain itself is a potent modulator of GI motility (Benato et al. 2019). This approach not only eliminates tissue trauma and postoperative inflammation but also removes the need for analgesic administration. Nevertheless, repeated handling, anaesthesia and radiographic procedures raise two related concerns: whether the 6‐day washout period was adequate to prevent physiological carry‐over effects and whether repeated procedures induced stress that could independently influence GI motility. Stress is a well‐established factor in GI stasis in rabbits, characterized by suppressed appetite and reduced GI motility (Lichtenberger and Lennox 2010). No formal behavioural assessments or objective physiological or biochemical stress markers, such as faecal glucocorticoid metabolites or heart rate variability, were employed in this investigation; consequently, we cannot definitively ascertain the animals’ stress levels during handling, anaesthesia or recovery. GI motility in rabbits exhibits a distinct diurnal variation, alternating between phases of hard faeces production and cecotrophy (Gidenne et al. 2010). To minimize the confounding effect of this circadian rhythm on transit time, it was imperative to initiate all experimental sessions at a strictly standardized time (10:00 AM). Given the demanding nature of the protocol—which involved anaesthesia followed by up to several hours of serial radiography—this temporal standardization restricted the experimental capacity to evaluating only one rabbit per day. Consequently, this temporal standardization resulted in a 6‐day interval between successive experimental conditions for each rabbit in the crossover design. From a pharmacokinetic standpoint, the 6‐day washout period seemed adequate, as isoflurane undergoes minimal biotransformation and is rapidly eliminated primarily through exhalation (Eger 1981; Flecknell 2009). In addition, daily feed intake and faecal output were monitored throughout the study, including during the adaptation period before any anaesthetic exposure. Repeated‐measures ANOVA showed no significant effects of condition (feed intake: *p* = 0.568; faecal output: *p* = 0.330), time (*p* = 0.184; *p* = 0.314) or their interaction (*p* = 0.388; *p* = 0.258) on either parameter. Only a single rabbit in the 60‐min group exhibited a transient reduction in feed intake (50 g compared with a group mean of 89.4 g [SD 22.8]) on the first post‐anaesthetic day, with spontaneous resolution thereafter. These findings did not reveal evidence of a persistent disturbance in GI function during the repeated experimental protocol; however, transient or subclinical effects from residual anaesthetic or stress cannot be entirely ruled out. Future studies incorporating validated behavioural and physiological stress markers are recommended to distinguish stress‐related effects from the direct effects of isoflurane on GI transit time.

This study has several important limitations. First, the small final sample size (*n* = 5) limited statistical power, particularly for multiple pairwise comparisons and for detecting potentially clinically significant effects. No formal a priori sample size calculation or power analysis was conducted prior to the study. Six rabbits were initially enrolled; however, one rabbit was excluded during the experimental phase following an unforeseen procedural incident, leaving five rabbits for the final statistical analysis. The number of animals was also constrained by the strict temporal standardization required to control for diurnal variation, which limited the experimental capacity to evaluate only one animal per day. Although the repeated‐measures design, in which each subject served as its own control, helped reduce inter‐individual variability, the results should be interpreted with caution. Future research should involve larger samples based on prospective power analyses to yield more accurate estimates and validate these findings. Second, the discrete ordinal nature of transit measurements, constrained to predefined radiographic intervals, contributed to substantial variability and reduced precision. More frequent radiographic intervals during critical time windows or alternative continuous monitoring methods, such as fluoroscopy or ultrasonography, would improve measurement precision and permit parametric statistical analysis. Third, only three anaesthetic durations (15, 30 and 60 min) were assessed; intermediate time points, such as 45 min, would better characterize the apparent threshold between 30 and 60 min, where differences were most pronounced. Fourth, the study population was restricted to healthy male rabbits. Healthy animals were intentionally selected as an initial model to evaluate the effects of isoflurane on GI function under standardized physiological conditions, eliminating potential confounders such as pre‐existing GI disease or systemic disorders. Only male rabbits were included because, in our pilot study, female rabbits exhibited hair‐pulling behaviour that could interfere with radiographic assessment and potentially affect GI transit time by ingestion of hair, irrespective of the anaesthetic administered. Although this behaviour occurs in both sexes (Reinhardt 2005), it was observed solely in female rabbits and did not encounter it in males. This approach ensured measurement reliability but restricts the generalizability. Fifth, only isoflurane was evaluated; isoflurane was selected for this initial investigation due to its widespread clinical use in rabbits and favourable pharmacokinetic profile, coupled with the absence of prior data regarding its GI effects in this species. Comparative studies with other anaesthetics, such as sevoflurane, would determine whether the findings are agent‐specific or represent class effects common to volatile anaesthetics. Finally, only heart rate, pulse rate, oxygen saturation (SpO_2_), respiratory rate and end‐tidal CO_2_ were monitored and used to ensure physiological stability. Future studies should incorporate blood pressure and body temperature monitoring for more comprehensive anaesthetic monitoring.

Despite these limitations, this study provides the first empirical assessment of isoflurane's effects on GI transit in rabbits. The findings establish a foundation for future adequately powered investigations in larger cohorts and clinically relevant populations. A fundamental consideration when interpreting these results is the distinction between statistical significance and clinical relevance. No significant differences were observed in gastric transit or time to hard faeces formation across the different durations of anaesthesia. Although a statistically significant difference was observed in small intestinal transit time, the absence of a significant difference between the anaesthesia groups and the awake control, together with the discrete nature of the radiographic measurements, limits its interpretation as evidence of a clinically relevant delay. Importantly, this finding was not accompanied by changes in daily feed intake, faecal output or time to hard faeces formation, and no clinically apparent GI dysfunction was observed under the experimental conditions studied. However, clinical applicability requires caution. Our study population consisted of young, healthy male rabbits undergoing non‐surgical anaesthesia. Clinical patients typically experience additional risk factors, including surgical trauma and inflammatory responses, opioid analgesics with known GI inhibitory effects, longer anaesthetic durations potentially exceeding 60 min, pre‐existing GI compromise or subclinical hypomotility and concurrent systemic disease. Each of these factors could potentiate isoflurane's effects or lower the threshold for clinical stasis. Although our findings did not demonstrate clinically evident impairment of feed intake or faecal output following isoflurane anaesthesia for up to 60 min under the conditions studied, clinicians should remain vigilant when multiple risk factors coexist and implement proactive monitoring of appetite and faecal output, particularly following prolonged anaesthetic episodes.

## 5 Conclusion

This study found no significant effects of isoflurane anaesthesia on gastric transit time, time to hard faeces formation, feed intake or faecal output in healthy male rabbits under the experimental conditions studied. Although an overall difference in small intestinal transit time was observed among conditions, no significant pairwise difference was detected between the anaesthesia groups and their awake (control) groups. Given the small sample size and the discrete nature of radiographic transit measurements, these findings should be interpreted cautiously. Larger studies using more precise methods of GI transit time assessment are warranted to determine whether isoflurane produces clinically meaningful alterations in GI motility in rabbits.

## Author Contributions

**Amir Zarein**: conceptualization, writing – original draft, methodology, validation, visualization, writing – review and editing, software, data curation. **Amir Rostami**: investigation, methodology, supervision, resources. **Majid Masoudifard**: data curation. **Saeed Farzad‐Mohajeri**: project administration. **Alireza Bahonar**: formal analysis. **Haniyeh Naghdi Liyeh**: writing – review and editing. **Zahra Sohani**: writing – review and editing.

## Funding

The authors have nothing to report.

## Ethics Statement

All procedures were approved by the Iranian Animal Ethics Committee (protocol number IR.UT.VETMED.REC.1404.046).

## Conflicts of Interest

The authors declare no conflicts of interest.

## Data Availability

The data that support the findings of this study are available from the corresponding author upon reasonable request.
